# Clinical outcomes of 1 + PRN and 3 + Q3M regimens of intravitreal conbercept injection for exudative age-related macular degeneration

**DOI:** 10.1038/s41598-020-65000-5

**Published:** 2020-05-14

**Authors:** Lei Gao, Jian Liu, Peng Zhang, Jianhua Ma, Hong Wang

**Affiliations:** 1 Department of Ophthalmology, Jinan 2nd People’s Hospital, Shandong Province, Jinan, 250001 China; 2 Department of Ophthalmology, Shandong Invalids General Hospital, Shandong Province, Jinan, 250001 China; 3 Department of Ophthalmology, Qilu Hospital, Shandong University, Shandong Province, Jinan, 250001 China

**Keywords:** Clinical trials, Molecular medicine

## Abstract

This retrospective study aimed to analyze the clinical outcomes of two regimens of intravitreal injections of conbercept [1+*pro re nata* (PRN) and 3 + Q3M] for the therapy of exudative age-related macular degeneration (AMD). In total, 105 eyes diagnosed with exudative AMD were enrolled. The eyes in the 1+PRN group (n = 51) received intravitreal injection of conbercept one time, followed by PRN retreatment. The eyes in the 3 + Q3M group (n = 54) received intravitreal injection of conbercept on three consecutive monthly, subsequently, once every three months for three times. After treatment, patients were followed up for 12 months. The best-corrected visual acuity (BCVA), central retinal thickness (CRT), and choroidal neovascularization (CNV) leakage area were compared before and after treatment. Moreover, the number of injections and adverse reactions were recorded. Compared with the 1+PRN group, BCVA was significantly improved and CRT was remarkably decreased in the 3 + Q3M group at 3, 6 and 12 months after operation. The disappeared or reduced CNV leakage area (93%) of the 3 + Q3M group was higher than that of the 1 + PRN group at the last follow-up. Moreover, the mean numbers of conbercept injections of the 1 + PRN group were less than the 3 + Q3M group. During the follow-up, there were no serious adverse reactions or ocular complications. This study reveals that intravitreal injection of conbercept using 3 + Q3M regimen has certain advantages than 1 + PRN regimen in extending drug delivery interval, improving patient’s vision, and reducing CRT.

## Introduction

Age-related macular degeneration (AMD) is one of the major causes of irreversible blindness in the elderly in the developing world ^1,2^. It is estimated that the prevalence of AMD among people 55 years of age and older is around 8.7% in the world^3^, and will increase in the near future since the proportion of the population over 65 years of age in developed countries is predicted to rise dramatically^4^. AMD can be divided into non-exudative and exudative types, which are also known as dry or non-neovascular and wet or neovascular AMD. Although only about 10% of AMD patients have the exudative form, the incidence of severe vision impairment and blindness is as high as 80%–90%^5^.

One of the pathological mechanisms of exudative AMD-caused visual loss is the presence of choroidal neovascularization (CNV), the formation of aberrant new vessels arising from pre-existing choroidal^6–8^. It is reported that vascular endothelial growth factor (VEGF) is a crucial regulator in CNV formation^9^ and is identified as the main target for exudative AMD treatment^10^. Large-scale clinical trials have confirmed that intravitreal injection of VEGF antagonists can prevent or improve vision loss in patients with exudative AMD ^11–13^.

Conbercept (KH902; Chengdu Kanghong Biotech Co., Ltd., Sichuan, China) is the first independently developed anti-VEGF drug in China, which is a fusion protein consisting of the VEGF binding domains of VEGF receptor 1 and 2 combined with the Fc portion of the human immunoglobulin G1, and has many characteristics such as multiple targets, strong affinity and long action time ^14,15^. A phase 1 study has shown that conbercept can improve best-corrected visual acuity (BCVA), reduce central retinal thickness (CRT), and decreased CNV area in patients with exudative AMD^16^. A phase 2 study has also confirmed that visual acuity and anatomic benefits are obtained from repeated intravitreal conbercept injections that are well tolerated in patients with exudative AMD during the 12 months^17^. Notably, conbercept was approved for the treatment of exudative AMD by China Food and Drug Administration in 2013 and was incorporated into the national basic medical insurance category B by the Ministry of Human Resources and Social Security of China on July 13, 2017, which significantly reduced the economic burden of patients. Although conbercept has been widely used in clinical practice, there is still no consensus on its treatment regimen and standards.

The present study retrospectively analyzed the clinical outcomes of two regimens of intravitreal injections of conbercept [1+ *pro re nata* (PRN) and 3 + Q3M] for the treatment of exudative AMD, thus providing a reference for further standardizing the clinical application of conbercept treatment.

## Results

### Baseline data

There was no significant difference in age, sex and baseline ocular characteristics between the two groups (P > 0. 05) (Table 1).

**Table 1:** Baseline characteristics of patients in the two groups.

Characteristics	1 + PRN (n = 51)	3 + Q3M (n = 54)	t/χ2	P
Sex			0.2410	0.6235
Male	27	26		
Female	24	28		
Age (years)	61.82 ± 6.73	60.94 ± 7.11	0.6505	0.5168
Eye			0.2587	0.6110
Right	26	27		
Left	25	27		
BCVA (LogMAR)	0.84 ± 0.38	0.81 ± 0.39	0.3989	0.6908
CRT (μm)	432.74 ± 69.81	425.38 ± 71.02	0.5351	0.5937
CNV Location			0.193	0.908
Extrafoveal	17(33.3%)	19(35.2%)		
Juxtafoveal	21(41.2%)	20(37.0%)		
Subfoveal	13(25.5%)	15(27.8%)		

### Visual acuity

The BCVA in the two groups before and after operation was compared (Table 2). The mean BCVA changed from baseline to 12 months was from 0.84 ± 0.38 to 0.50 ± 0.25, and from 0.81 ± 0.39 to 0.33 ± 0.23 LogMAR in the 1 + PRN and 3 + Q3M groups, respectively. Before operation, no statistically significant difference in BCVA existed between the two groups (P > 0.05). In each group, significant difference in BCVA existed between before and after operation (P < 0.05). Moreover, at 3, 6 and 12 months after operation, BCVA was significantly improved in the 3 + Q3M group compared with that in the 1 + PRN group (P < 0.05).

**Table 2:** Best-corrected visual acuity (LogMAR) in the two groups before and after treatment.

Groups	Before operation	1 month	3 month	6 month	12 month
		after operation			
1 + PRN (n = 51)	0.84 ± 0.38	0.54 ± 0.32	0.50 ± 0.29	0.48 ± 0.26	0.50 ± 0.25
3 + Q3M (n = 54)	0.81 ± 0.39	0.52 ± 0.31	0.36 ± 0.26	0.31 ± 0.24	0.33 ± 0.23*
t	0.3989	0.3253	2.6075	3.4838	3.6289
P	0.6908	0.7456	0.0105	0.0007	0.0004

*Compared to 1 + PRN group at each time of follow-up.

#### CRT

The CRT in the two groups before and after operation was shown in Table 3. The mean CRT was decreased from 432.74 ± 69.81 to 256.34 ± 40.22 μm in the 1 + PRN group, and from 425.38 ± 71.02 to 235.91 ± 38.03 μm in the 3 + Q3M group. There was no significant difference in CRT between 1 + PRN and 3 + Q3M groups before operation (P = 0.5937). However, the average CRT was remarkably decreased in the 3 + Q3M group relative to that in the 1 + PRN group after 3, 6 and 12 months of operation (P < 0.05).

**Table 3:** Central retinal thickness (μm) in the two groups before and after treatment.

Groups	Before treatment	1 month	3 month	6 month	12 month
		after treatmentn			
1 + PRN (n = 51)	432.74 ± 69.81	286.94 ± 54.27	266.53 ± 40.07	258.15 ± 40.03	256.34 ± 40.22
3 + Q3M (n = 54)	425.38 ± 71.02	279.87 ± 57.62	239.17 ± 37.71	233.01 ± 37.86	235.91 ± 38.03
t	0.5351	0.6464	3.6045	3.3074	2.67542
P	0.5937	0.5159	0.0005	0.0013	0.0087

*compared to 1 + PRN group at each time of follow-up.

### CNV leakage

At the last follow-up, FFA and ICGA examination showed that, in the 1 + PRN group, the leakage area of CNV disappeared in 26 eyes (51%), reduced in 18 eyes (35%) and enlarged in 7 eyes (14%); in the 3 + Q3M group, the leakage area of CNV disappeared in 32 eyes (60%), reduced in 18 eyes (33%) and enlarged in 4 eyes (7%).

### Number of injections

The mean numbers of conbercept injections of 51 eyes in the 1 + PRN group were 5.14 ± 0.93. Among them, 7 eyes were injected for 3 times, 12 eyes for 4 times, 26 eyes for 5 times, and 6 eyes for 6 times. The 54 eyes in the 3 + Q3M group were all injected for 6 times. There was significant different difference in number of injections between the two groups (t = 6.6039, P < 0.001).

### Adverse events

In terms of adverse events associated with injection and drug, some patients in the two groups had foreign body sensation and water circle fluttering in front of the eye, without other special discomfort. During the follow-up, there were no severe ocular complications related to treatment, such as retinal detachment, retinal tear, continuously elevated intraocular pressure, endophthalmitis. Moreover, no serious adverse reactions are observed.

## Discussion

With the aging of the population and the improvement of socioeconomic conditions, the prevalence of AMD is also gradually rising among the Chinese population^18^. Intravitreal injection of anti-VEGF drugs has been considered as the most effective treatment for exudative AMD ^19,20^. However, for the majority of elderly patients, poor compliance and frequent treatment impose a financial burden on them. In addition, frequent intravitreal injections may increase the risk of endophthalmitis and other adverse reactions^21^, and the number of injections is also related to the development of geographic atrophy of macular region ^22,23^. In China, frequent intravitreal injections have been applied to treat patients with exudative AMD. Therefore, exploration of standardized treatment regimen that can reduce the frequency of intravitreal injections and ensure the therapeutic effect has become the current research hotspot of anti-VEGF treatment.

The investigation of optimal treatment regimens of intravitreal injection of conbercept has never stopped. A randomized double-mask, multicenter study has revealed that significant gains in BCVA are obtained after intravitreal conbercept injections for 3 consecutive months, and subsequently monthly (Q1M) or PRN treatments with conbercept are also safe and well tolerated during the 12 months^17^, indicating that different treatment regimens during the prolonging the treatment period can maintain stable curative effect and meet personalized treatment requirements according to different conditions in clinical practice. Moreover, a recent data from the phase three clinical trial of conbercept has also confirmed that the 3 + Q3M treatment regimen is safe and effective for the treatment of exudative AMD^24^. However, in clinical practice, because elderly patients have poorer adherence and less awareness of the characteristics of exudative AMD, they often ignore the later reexamination after obtaining good visual acuity after the initial injection, and some patients receive repeated injection treatment only when their vision drops again. Therefore, more patients adopt the 1 + PRN treatment regimen. Previous studies have shown that any delays in intravitreal injection of anti-VEGF drugs may result in vision benefit impairment or even vision loss ^12,25,26^. Therefore, we retrospectively analyzed the clinical outcomes of 1 + PRN and 3 + Q3M in the treatment of exudative AMD.

In the present study, the mean BCVA changed from baseline to 12 months was from 0.84 ± 0.38 to 0.50 ± 0.25, and from 0.81 ± 0.39 to 0.33 ± 0.23 LogMAR in the 1 + PRN and 3 + Q3M groups, respectively. The mean CRT was decreased from 432.74 ± 69.81 to 256.34 ± 40.22 μm in the 1 + PRN group, and from 425.38 ± 71.02 to 235.91 ± 38.03 μm in the 3 + Q3M group. These data were in line with previous findings that intravitreal injection of conbercept could improve visual acuity in patients with neovascular AMD ^27,28^. Moreover, CNV leakage area was remarkably disappeared or reduced in the 1 + PRN and 3 + Q3M groups, which was also consistent with previous reports that intravitreal administration of conbercept could prevent leakage of CNV in a nonhuman primate model ^29,30^. Therefore, we speculate that 1 + PRN and 3 + Q3M are all effective regimens for the treatment of exudative AMD. Furthermore, compared with the 1 + PRN group, BCVA was significantly increased and CRT was remarkably dcreased in the 3 + Q3M group at 3, 6 and 12 months after operation. The disappeared or reduced CNV leakage area (93%) of the 3 + Q3M group was higher than that of the 1 + PRN group (86%) at the last follow-up. It can therefore be speculated that 3 + Q3M is more effective in improving visual acuity, reducing CRT and reducing the CNV leakage area.

Furthermore, VEGF is shown to be an essential factor for the survival and maintenance of retinal pigment epithelium (RPE) integrity^31^. Intravitreal injections of anti-VEGF drugs too frequently is likely to increase the risk of RPE atrophy and choriocapillary atrophy ^32,33^. In addition, intravitreal injections of anti-VEGF agents too frequently may also result in systemic adverse events or ocular complications, such as endophthalmitis, acute elevation of systemic blood pressure, and vitreous hemorrhage^34^ or even lead to systemic thrombotic events^35^. Therefore, it is important to explore the treatment options that will allow effective treatment with minimal injections. In this study, the mean numbers of conbercept injections of the 1 + PRN group were significantly less than the 3 + Q3M group (5.14 ± 0.93 vs. 6, P < 0. 001). Due to the short follow-up times in this study, 13% of patients in the 1 + PRN group at 12months still had enlarged CNV leakage area, which needed to be repeated administration. With the extension of follow-up times, the number of injections of 3 + Q3M may be less than 1 + PRN, which requires to be further confirmed by more long-term studies.

Notably, the mechanism related to the systemic exposure to anti-VEGF drugs after intravitreal injection is very complex. Li *et al*. reported that the larger molecular size (143 kD) of conbercept might limit its permeability into blood-ocular barriers. Comparing with the systemic medication vitreous injection of conbercept probably reduced toxicity and decreasing the incidence of systemic adverse reactions^36^. In this study, there were no severe ocular complication during the follow-up, such as endophthalmitis, tractional retinal detachments, and elevated intraocular pressure. Besides, no serious systemic adverse reactions (eg. acute elevation of systemic blood pressure) occurred, which might be due to the small sample size and short follow-up time. Due to lack of clinical time in China, long-term efficacy and complications of conbercept have not been fully understood, requiring further in-depth clinical studies to investigate the conbercept efficacy and safety for the treatment of exudative AMD.

In conclusion, this study preliminarily reveals that 1 + PRN and 3 + Q3M are all effective regimens for the treatment of exudative AMD. Intravitreal injection of conbercept using 3 + Q3M regimen may have certain advantages in extending the drug delivery interval, improving the patient’s vision and reducing the CRT. These findings will provide a reference for the standardized treatment of exudative AMD.

## Materials and methods

### Patients

This retrospective study included a total of 105 eyes from 105 patients who were diagnosed with exudative AMD using fundus fluorescein angiography (FFA) and indocyanine green angiography (ICGA) in the Department of Ophthalmology, Qilu Hospital of Shandong University from January 2015 to August 2018. There were 53 males (53 eyes) and 52 females (52 eyes), aged from 50 to 83 (mean 65.8) years old.

All patients were subjected to ophthalmoscopic examination, including BCVA measurement, intraocular pressure, color fundus photography, FFA, ICGA and optical coherence tomography (OCT). BCVA was checked using the international standard logarithmic visual acuity chart, and the logMAR notation was recorded. The intraocular pressure was measured by non-contact tonometer (Topcon, Tokyo, Japan). Color fundus photography, FFA, and ICGA was detected by Topcon Fundus Camera (Tokyo, Japan); OCT was evaluated by Heidelberg Engineering (Heidelberg, Germany).

The inclusion criteria were as follows: (1) age ≥ 50 years; (2) AMD-induced any type of choroidal neovascularization (CNV) under or near the fovea diagnosed by FFA and ICGA examination; and (3) follow-up according to the treatment plan. Exclusion criteria were: (1) previous intravitreal injection of anti-VEGF drugs or receiving laser photocoagulation therapy; (2) previous history of ocular surgery other than cataract; (3) CNV caused by other reasons; (4) combined with diabetic retinopathy and other retinal diseases;(5) combined with severe systemic diseases affecting intravitreal injection; and (6) severe effects of refractive stromal unclear.

This study was conducted after approval by the Ethics Committee of Qilu Hospital of Shandong University, and written informed consent was provided by all patients after being aware of possible treatment risks. Besides, all experiments in our study adhered to the tenets of the Declaration of Helsinki.

### Surgical procedures

All the eyes received intravitreal injection of conbercept (0.5 mg/0.05 mL, Chengdu Kanghong Biotechnology Co. Ltd., Chengdu, China) by the same senior physician as previously described^37^. From three days before surgery, 0.5% levofloxacin eye drops were given, four times a day. All patients were divided into two groups: 1 + PRN (n = 51) and 3 + Q3M (n = 54). The eyes in the 1 + PRN group received intravitreal injection of conbercept one time, followed by PRN retreatment if one of the following changes were observed as previously described:^37,38^ recurrent, or persistent subretinal or intraretinal fluid based on the OCT scan; new macular hemorrhage; new leakage or new onset of classic CNV on FFA; and vision loss> one line or conscious vision decrease. The eyes in the 3 + Q3M group received intravitreal injection of conbercept on three consecutive monthly, subsequently, the eyes received intravitreal injection of conbercept once every three months for three times. The injection sites on the conjunctival capsule were coated with tobramycin dexamethasone ointment, bandage eyes. The next day after surgery, 0.5% levofloxacin eye drops were given, four times a day for three consecutive days.

### Follow-up

Patients were followed up for 12 months after operation. Routine visual acuity, intraocular pressure and OCT examination were performed every month. FFA examination was conducted every 3 months. The changes of BCVA, CRT, and the leakage area of CNV before and after operation were observed in all patients. Moreover, the adverse reactions were recorded. All experiments were performed in accordance with relevant guidelines and regulations.

### Statistical analysis

Statistical analysis was con*ducte*d using SPSS 22.0 statistical software. The measurement data were expressed as mean ± standard deviation (SD). The differences between the two groups at each time point were compared using independent sample t test. The differences between different time points in each group were analyzed by Fisher’s least significant difference (LSD) test. P < 0.05 indicated a statistically significant result.
